# Pediatric Patients With Steroid-Sensitive Nephrotic Syndrome Have Higher Expression of T Regulatory Lymphocytes in Comparison to Steroid-Resistant Disease

**DOI:** 10.3389/fped.2019.00114

**Published:** 2019-04-02

**Authors:** Fabio Tadeu Lourenço Guimarães, Rodrigo Novaes Ferreira, Gustavo Eustáquio Alvim Brito-Melo, Etel Rocha-Vieira, Wagner de Fátima Pereira, Sérgio Veloso Brant Pinheiro, Aline Silva Miranda, Ana Cristina Simões e Silva

**Affiliations:** ^1^Centro Integrado de Pós-Graduação e Pesquisa em Saúde - CIPq, Universidade Federal dos Vales do Jequitinhonha e Mucuri (UFVJM), Diamantina, Brazil; ^2^Departamento de Morfologia, Instituto de Ciências Biológicas, Universidade Federal de Minas Gerais (UFMG), Belo Horizonte, Brazil; ^3^Unidade de Nefrologia Pediátrica, Departamento de Pediatria, Faculdade de Medicina, Universidade Federal de Minas Gerais (UFMG), Belo Horizonte, Brazil; ^4^Laboratório Interdisciplinar de Investigação Médica, Faculdade de Medicina, Universidade Federal de Minas Gerais (UFMG), Belo Horizonte, Brazil

**Keywords:** nephrotic syndrome, flow cytometry, regulatory T cells, integrins, chemokines

## Abstract

**Background and Aim:** Idiopathic nephrotic syndrome (INS) is classified according to the response to drug therapy in steroid-sensitive (SS), steroid-dependent (SD), and steroid-resistant (SR) categories. Previous studies showed changes in inflammatory activity of subpopulations of lymphocytes in INS. This study aimed to compare SS and SR patients in regard to subpopulations of leukocytes, profile of regulatory lymphocytes, and migratory activity of lymphocyte subpopulations. Results obtained in INS patients were also compared to age and sex-matched healthy controls.

**Methods:** This is a cross-sectional study including SS patients (*n* = 30), SR patients (*n* = 14), and controls (*n* = 10). Peripheral blood samples were withdrawn for *ex-vivo* leukocyte flow cytometry analysis.

**Results:** Percentage of B-lymphocytes and natural killer (NK) cells were significantly reduced in SR patients when compared to controls, while the percentage of NKT cells were decreased in SS patients in comparison to controls. Percentages of CD4^+^ expressing FoxP3 and CTLA4 were significantly higher in SS patients in comparison to SR patients and controls. The expression of integrin CD18 on the surface of T lymphocytes (CD3^+^) was reduced in SS patients if compared to controls.

**Conclusion:** This study found that SS INS patients have higher levels of regulatory T-lymphocytes and lower expression of adhesion molecules than SR patients.

## Introduction

Nephrotic syndrome is a glomerular disease, highly prevalent in children, and characterized by a combination of intense proteinuria, hypoproteinemia, and edema (1–4). Nephrotic syndrome can be secondary to other diseases or, more commonly, due to primary changes in the kidney, not fully elucidated, and named as idiopathic nephrotic syndrome (INS). The initial treatment of INS is based on a course of oral corticosteroids. The response to corticosteroids therapy is still the most important prognostic factor in INS (2). According to the response to steroids, patients are classified as steroid-sensitive (SS), steroid-dependent (SD), and steroid-resistant (SR) (2, 3).

The response to steroids or to other immunosuppressive mediations is a strong evidence for the immune nature of INS. However, cellular and molecular mechanisms underlying INS pathophysiology remain to be fully elucidated (5, 6). Several studies support a role for T lymphocytes subpopulations CD8^+^ (7, 8), and CD4^+^ (6, 9, 10), for cytokine/chemokine release including IL-2, IL-10 (10), IL- 4 (11), and IL-8 (12, 13), for the complement system (14), macrophages (15–17), and NK cells (18) in INS.

Tissue homeostasis relies on the balance between inflammatory and anti-inflammatory responses. There is evidence that some chronic degenerative based-inflammatory disorders might be associated with a dysfunction in immune-regulatory mechanisms rather than an excessive activation of the immune response. For instance, a decrease in the percentage of TCD4^+^CD25^High+^FoxP3^+^ lymphocytes, classically known as T regulatory cells (T_regs_), was reported in patients with autoimmune diseases, including rheumatoid arthritis, systemic lupus erythematous, vascular injury mediated by anti-neutrophil cytoplasmic antibodies, inflammatory myopathies, and systemic sclerosis (19).

Current advances in the knowledge of regulatory mechanisms have revealed that the control or regulation of the immune response is a complex process not limited to the presence or absence of TCD4^+^CD25^High+^FoxP3^+^ cells. The immune regulatory response can also include the expression of inhibitory molecules, activation of cell migration, and intercellular cooperation network. Accordingly, the cytotoxic T-lymphocyte-associated protein 4 (CTLA-4) molecule, by interacting with the CD80 and CD86 ligands, inhibits cell proliferation, and cytokine production, which, in turn, contribute to regulatory functions exerted by T_reg_ cells (20–22). It has also been reported that NKT cells may assist T_reg_ lymphocytes activity (23).

The receptor LFA-1 (Lymphocyte function-associated antigen 1), also termed CD18, is a protein member of integrin family responsible for the recruitment of cells to injury or inflammation sites (24, 25). This molecule is expressed on the surface of activated T-lymphocytes and enhances the interaction between T cells and antigen presenting cells (25). It was previously reported that CD3^+^ cells expressing CD18 were increased in renal tissue of pediatric patients with INS (16). However, the expression of CD18 in subpopulations of lymphocytes, helper, and cytotoxic, has not been investigated in peripheral blood of pediatric patients with INS.

Recently, we have shown that INS patients presented have expression of the inflammatory cytokine TNF in TCD4^+^-lymphocyte cells. Interestingly, a more intense inflammatory response was found during relapses of proteinuria (26). Considering that inflammatory disorders seem to be also associated with dysfunctions in immune-regulatory mechanisms, this study aimed to compare SS and SR patients in regard to subpopulations of leukocytes, profile of regulatory lymphocytes, and migratory activity of lymphocyte subpopulations. Results obtained in INS patients were also compared to age and sex-matched healthy controls. We hypothesized that the concentration of subpopulations of lymphocytes and the expression of regulatory and adhesion molecules may differ in SS and SR INS patients.

## Patients and Methods

The current cross-sectional study was conducted using a sample of 44 children with INS and 10 healthy sex and age-matched children as a control group. The sample of patients and controls included in this study was previously analyzed in order to investigate the expression of T-lymphocytes-associated cytokines in patients during relapses in comparison to patients in remission (26). Therefore, study protocol, inclusion and exclusion criteria for INS patients and healthy controls and ethical issues were the same as previously described in more detail (26).

Briefly, inclusion criteria included pediatric patients with well-established INS and still-preserved renal function, whose parents gave their consent to participate in the study protocol. Congenital or secondary forms of nephrotic syndrome and INS patients with estimated glomerular filtration rate below 90 mL/min/1.73 m^2^ were excluded from the study. INS patients were classified in two subgroups based on the response to corticosteroid therapy: steroid-sensitive (SS, *n* = 25, 16 boys and 9 girls) and steroid-resistant patients (SR, *n* = 14, 8 boys, and 6 girls) and were recruited from the Pediatric Nephrology Unit of our institution.

The control group comprised of healthy subjects randomly recruited from the Pediatric Primary Care Center of our institution. It happened that the mean value of the age group, the gender distribution and the percentage of white and non-white individuals did not differ in controls and INS patients. Controls were also from the same geographic area and with undistinguished socioeconomic conditions in comparison to patients. Healthy status was determined through the subjects' medical history and either a parental report or self-report to exclude the presence of chronic or acute diseases.

The Ethics Committee of the institution approved the study. Informed consent was obtained from parents of all included patients and healthy controls. The research protocol did not interfere with any medical recommendations or prescriptions. The follow-up of the INS patients and healthy controls was guaranteed even in cases of refusal to participate in the study.

The diagnostic criteria for INS were based on *International Study of Kidney Disease in Children* (27). Our pediatric patients with nephrotic syndrome were followed up according to a well-established protocol, which included investigation of disease etiology, assessment of clinical course and laboratory alterations, institution of treatment, and indication for renal biopsy based on clinical (corticosteroid unresponsiveness) and laboratory findings. After establishment of the diagnosis of INS, patients received daily prednisone or prednisolone 60 mg/m^2^ for at least 6 weeks, followed by further doses administered on alternate days for 4–6 weeks. Then, prednisone/prednisolone was progressively tapered off at a rate of 25% every 2 weeks until complete discontinuation by the fourth month. Patients who achieved complete remission of edema and proteinuria during the first 8 weeks of steroid therapy were considered SS, whereas those who did not respond to steroids after 8 weeks of administration were defined as SR and were given oral cyclosporine A at an initial daily dose of 5 mg/kg for at least 2 years after complete remission of nephrotic syndrome. The dose of cyclosporine was subsequently adjusted to achieve blood levels between 80 and 200 ng/ml at the C0 point. Angiotensin converting enzyme (ACE) inhibitors and/or angiotensin receptor blockers were used to help controlling proteinuria.

Peripheral blood samples were collected into sterile vacuum tubes containing EDTA and were used for biochemical testing and *ex-vivo* phenotypic characterization of circulating leukocytes by flow cytometry. Biochemical tests were processed within 30 min after sampling to measure total cholesterol, triglycerides, urea, creatinine, and albumin. Phenotypic analysis was processed within 24 h. Cells were pelleted by centrifugation at 700 × g for 10 min at 4°C and plasma was collected and re-centrifuged (1,300 × g for 20 min) (26). Twenty-four-hour urine samples were collected to measure proteinuria by standard methods in all patients at the same time-point of blood sampling for flow cytometry. Glomerular filtration rate (GFR) was estimated by the modified Schwartz formula (28).

Cell surface markers *ex-vivo* analysis of peripheral blood leukocytes was performed using the immunofluorescence method recommended by Becton Dickinson (BD, San Diego, CA—USA) and modified as follows: 50 microliters (μL) of blood were incubated in the dark for 30 min at room temperature (RT) with 1–5 μL of monoclonal antibodies mixture specific for cell surface markers, conjugated to the fluorochromes Fluorescein isothiocyanate -FITC and phycoerythrin-PE.

Antibodies used were anti-CD3 PE (clone: HIT3a), anti-CD18 PE (clone: 6.7), anti-CD56 PE (clone: B159) from BD Biosciences; anti-CD4 FITC (clone: RPA-T4), anti-CD8 PE (clone: HIT8a), anti-CD19 FITC (clone: HIB19), from BD Pharmingen^tm^; anti-CTLA4 PE (clone: 1493), anti-CD3 FITC (clone: UCHT1), anti-CD8 FITC clone: (HIT8a) from BioLegend.

After incubation erythrocytes were lysed with 2 mL of RBC lysis solution (100 μL lysis solution—Optilyse-B, Immunotec, USA) and incubated in the dark for 10 min at RT, followed by centrifugation at RT protected from light. Then, the cells were washed twice with 1 mL of phosphate buffered saline (PBS = 0.15 mol/L sodium chloride, 0.01 mol/L sodium phosphate—pH 7.2–7.4).

Intracytoplasmic staining of cells was performed with an addition of PBS containing 0.5% bovine serum albumin; 0.1% sodium azide and 0.5% saponin—pH 7.2 at 7.4 for 10 min at RT, following incubation with the anti-FOXP3-PE monoclonal antibody (clone: 259d/C7, BD Biosciences) for 30 min. After incubation period, the cells were washed again with permeabilizing solution. The samples were immediately analyzed on a flow cytometer.

Data acquisition was performed using a flow cytometry FACScan model (Becton-Dickinson, USA). Ten thousand events for data analysis were acquired using the Cell Quest software. Graphics of point distribution depending on size (FSC) and granulosity (SSC) of cell, followed by marking analysis with fluorescent antibodies were used to identification of cell populations. These cells were then analyzed for their expression [frequency and mean fluorescent intensity (MFI)] of a given marker using histograms with markers set based on negative isotype controls.

Frequencies of B lymphocytes (CD3^−^CD19^+^), cells with suggestive phenotype of NK lymphocytes (CD3^−^CD56^+^), NKT (CD3^+^CD56^+^), and the percentage of T CD4^+^ cells expressing CTLA4 were evaluated in phenotypic analysis of peripheral white blood cells of children with INS. Analysis by MFI was used to determine the expression of regulatory T cells (CD4^+^FoxP3^+^), CD18 integrin in T lymphocytes (CD3^+^CD18^+^), in T helper lymphocytes (CD4^+^CD18^+^), and in T cytotoxic lymphocytes (CD8^+^CD18^+^).

### Statistical Analysis

Results were shown as mean ± standard deviation, median, and interquartile ranges or percentages, when appropriate. All data were tested for normality by the Shapiro-Wilk test. For variables normally distributed, differences were compared by analysis of variance (ANOVA) followed by Bonferroni post-test for multiple comparisons. Unpaired Student *t*-test was used to compare two groups presenting variables with Gaussian distribution. In case of variables with non-Gaussian distribution, differences were analyzed by Kruskal-Wallis non-parametric test followed by Dunn post-test or Mann Whitney test. Percentages were compared by chi-square test. All statistical tests were two tailed and the level of significance was set as *p* < 0.05. Statistical analyses were performed using SPSS software version 22.0 (SPSS Inc., Chicago, IL, USA) and GraphPad Prism version 5.0 (GraphPad Software, Inc., La Jolla, California, USA).

## Results

### Clinical Parameters

Patient's clinical data including demographic data, renal function parameters, biochemical analysis, medication in use and proteinuria are shown in Table 1. Patients with INS were divided in SS and SR according to response to steroids. The time since the diagnosis of INS was similar in both subgroups, being of 5.2 ± 2.3 years for SS patients and 5.5 ± 2.6 years for SR group. On the other hand, the number of relapses since diagnosis was higher in SR than SS patients (3.5 ± 1.3 vs. 1.7 ± 0.8 per year since diagnosis, *p* = 0.01). No significant differences were observed in the comparison between SS and SR groups regarding to age, gender distribution, race, plasma levels of urea, creatinine, triglycerides, cholesterol, and albumin. However, 24-h urinary protein excretion and protein/creatinine ratio were significantly higher in SR then in SS patients (Table 1). All INS patients had still normal glomerular filtration rate at the time of blood sampling. Total steroid dose was significantly higher in SR than in SS (1567.2 ± 238.6 mg/Kg vs. 387.4 ± 123.5 mg/Kg, *p* < 0.01). In regard to disease activity, all patients of the SS group exhibited total remission and were not receiving steroids and/or other immunosuppressive mediations for at least 6 months at the time of blood sampling. On the other hand, half of the patients with steroid resistance were in partial remission of the disease (*p* = 0.01, Table 1). Partial remission was considered when proteinuria decreased by at least 50% and below the nephrotic range cutoff, but did not normalize following a defined course of treatment. All SR patients were receiving cyclosporine A in combination with low steroid doses or angiotensin converting enzyme inhibitors for at least 2 years at the time of blood sampling. Our INS patients did not receive other medications as rituximab and cytoxan. As expected, medications in use differed significantly when SS and SR patients were compared (*p* < 0.001, Table 1).

**Table 1:** Clinical and biochemical feature of patients with idiopathic nephrotic syndrome sub-divided according to the therapeutic response to steroids.

**Parameters**	**SS (*n* = 25)**	**SR (*n* = 14)**	***P*-value**
Age (years)	11.2 ± 0.9	11.6 ± 1.0	0.79
Gender (male, %)	64%	57%	0.52
Race (white, %)	72%	71.4%	0.95
Urea (mg/dL)	25.8 ± 6.6	24.4 ± 5.0	0.44
Creatinine (mg/dL)	0.49 ± 0.1	0.46 ± 0.2	0.51
Triglycerides (mg/dL)	100 ± 58	99 ± 54	0.89
Total cholesterol (mg/dL)	162 ± 37	186 ± 47	0.14
Albumin (g/dL)	4.1 ± 1.0	3.6 ± 0.9	0.22
Glomerular filtration rate (ml/min)^†^	142.4 ± 11.3	141.9 ± 10.0	0.65
Proteinuria (mg/dL/24 h)	52 [10–78]	154 [60–690]	0.01
Protein/Creatinine ratio	0.05 [0.01–0.18]	0.45 [0.15–0.68]	<0.001
**DISEASE ACTIVITY**			
Complete remission	25 (100%)	6 (43%)	0.001
Partial remission	0	7 (50%)	–
Relapsed disease	0	1 (7%)	–
**MEDICATIONS**			
No medication	20 (80%)	0	<0.001
Only prednisone	0	0	–
Only ACE inhibitor	5 (20%)	0	–
Prednisone+Ciclosporine	0	9 (64%)	–
Ciclosporine+ACEi	0	5 (36%)	–

*Values expressed as mean ± standard deviation, median and interquartile range [25th percentile−75th percentile], individual values and percentages*.

† *Glomerular filtrations rate was estimated using the modified Schwartz formula (29). Means were compared by unpaired Student t-test, Mann-Whitney test was used to compare medians and chi-square test to compare percentages. SS, steroid-sensitive; SR, steroid-resistant; ACE, angiotensin converting enzyme; ACEi, angiotensin converting enzyme inhibitor*.

The control group did not differ in regards to the mean value of age (11.4 ± 1.2 years-old), gender distribution (60% of male), and race (70% of white) when compared to SS (11.2 ± 0.9 years-old, 64% of males and 72% of white individuals) and SR (11.6 ± 1.0 years-old, 57% of males, and 71.4% of white individuals) patients (*p* > 0.05 for all group comparisons). In addition, control individuals were from the same geographic area and with similar socioeconomic conditions. Medical history and physical examination ruled out any chronic or acute condition or use of medication at the time of blood sampling.

### Phenotype Analysis of Peripheral Blood Leukocytes in INS Patients

**Figure 1:**
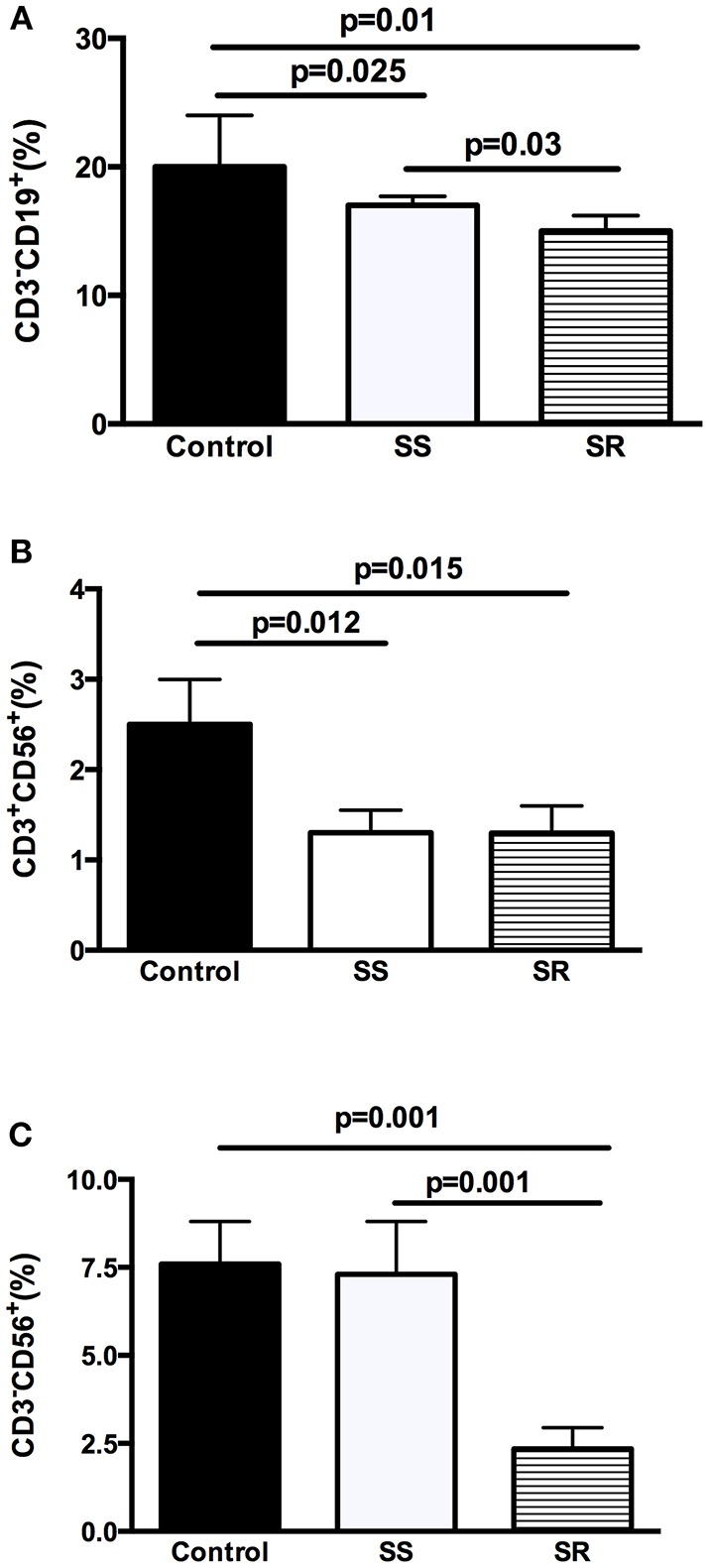
Analysis of cellular balance in peripheral blood lymphocytes of health controls and children with INS steroid-sensitive (SS) and steroid-resistant (SR): **(A)** percentage of B-lymphocytes (CD3^−^CD19^+^), **(B)** NKT (CD3^+^CD56^+^), and **(C)** NK (CD3^−^CD56^+^) suggestive cells. Results are expressed as bar graphs with mean values and standard deviation. *P*-values are displayed above lines that indicate each comparison.

Phenotype analysis revealed a significant lower percentage of B lymphocyte (CD3^−^CD19^+^) and NKT cells (CD3^+^CD56^+^) in both SS and SR groups if compared to controls (Figures 1A,B). As shown in Figure 1A, the percentage of CD3^−^CD19^+^ was of 20 ± 4% in controls in comparison to 17 ± 0.7% in SS patients (*p* = 0.025) and to 15 ± 1% in SR group (*p* = 0.01). The percentage of CD3^−^CD19^+^ was significantly lower in SR than in SS patients (15 ± 1% vs. 17 ± 0.7%, *p* = 0.03, Figure 1A). The percentage of CD3^+^CD56^+^ (2.5 ± 0.5%) was higher in controls when compared to SS (1.3 ± 0.3%, *p* = 0.012) and SR (1.3 ± 0.4%, *p* = 0.015) patients (Figure 1B). SS and SR groups had similar percentages of CD3^+^CD56^+^ (Figure 1B). As shown in Figure 1C, the percentage of NK (CD3^−^CD56^+^) cells was significantly lower in SR patients (2.4 ± 0.6%) in comparison to SS group (7.3 ± 1.5%, *p* = 0.001) and to controls (7.6 ± 1.2%, *p* = 0.01). SS patients and control group had similar percentages of CD3^+^CD56^+^ (Figure 1C).

### T_reg_ Lymphocytes (CD4^+^FoxP3^+^ and CD4^+^CTLA4^+^) Analysis in the Peripheral Blood of INS Patients

**Figure 2:**
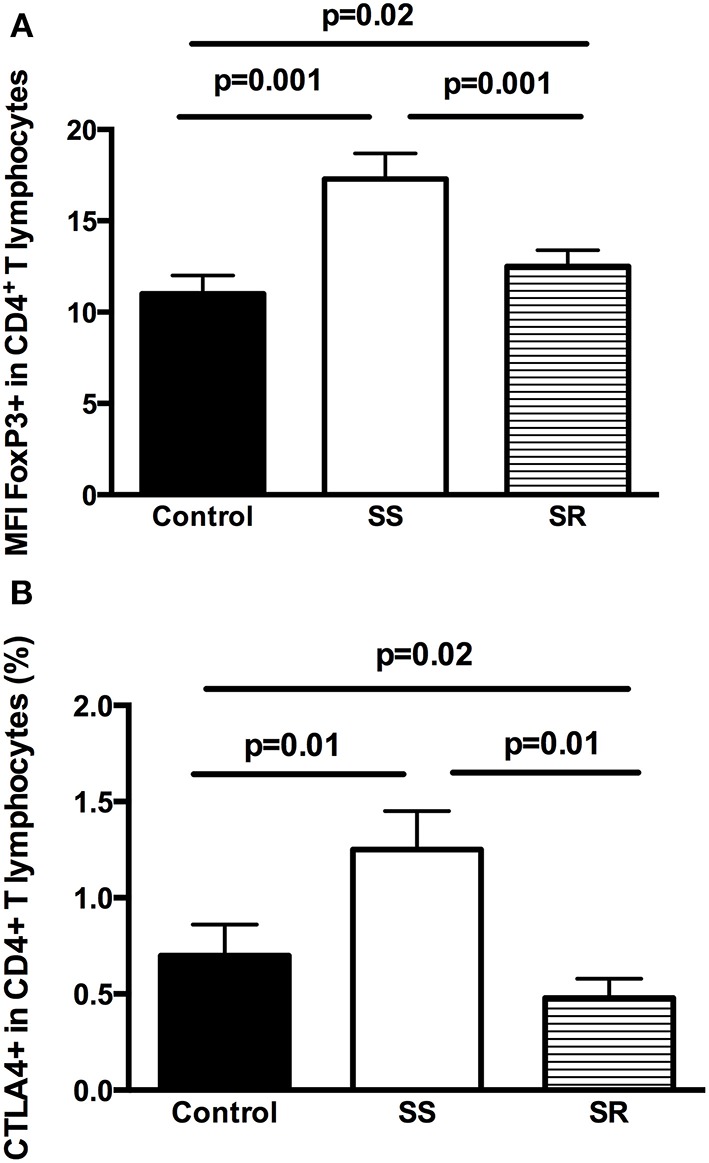
Analysis of regulatory T cells in peripheral blood lymphocytes of health controls and children with INS steroid-sensitive (SS) and steroid-resistant (SR): **(A)** TCD4^+^FoxP3^+^ lymphocytes and **(B)** TCD4^+^CTLA-4^+^ lymphocytes. Results are expressed as bar graphs with mean values and standard deviation. *P*-values are displayed above lines that indicate each comparison.

As shown in Figure 2A, the percentage of TCD4^+^ cells expressing FoxP3 was significantly higher in both SS (17.3 ± 1.4%) and SR groups (12.5 ± 0.9%) if compared to control group (*p* = 0.001 compared to SS and *p* = 0.02 compared to SR patients). Interestingly, the SR group had lower percentage of CD4^+^FoxP3^+^ T lymphocytes in comparison to SS group (12.5 ± 0.9% vs. 17.3 ± 1.4%, *p* = 0.001, Figure 2A).

Regarding the percentage of CD4^+^CTL4A4^+^ cells, SS patients had higher percentage when compared to controls (1.25 ± 0.20% vs. 0.70 ± 0.20%, *p* = 0.01) and to the SR group (1.25 ± 0.20% vs. 0.48 ± 0.10%, *p* = 0.01). Moreover, the percentage of these cells was lower in SR patients in comparison to control group (0.48 ± 0.10% vs. 0.70 ± 0.20%, *p* = 0.02, Figure 2B).

### Migration Profile Analysis of Peripheral Blood Lymphocytes in INS Patients

**Figure 3:**
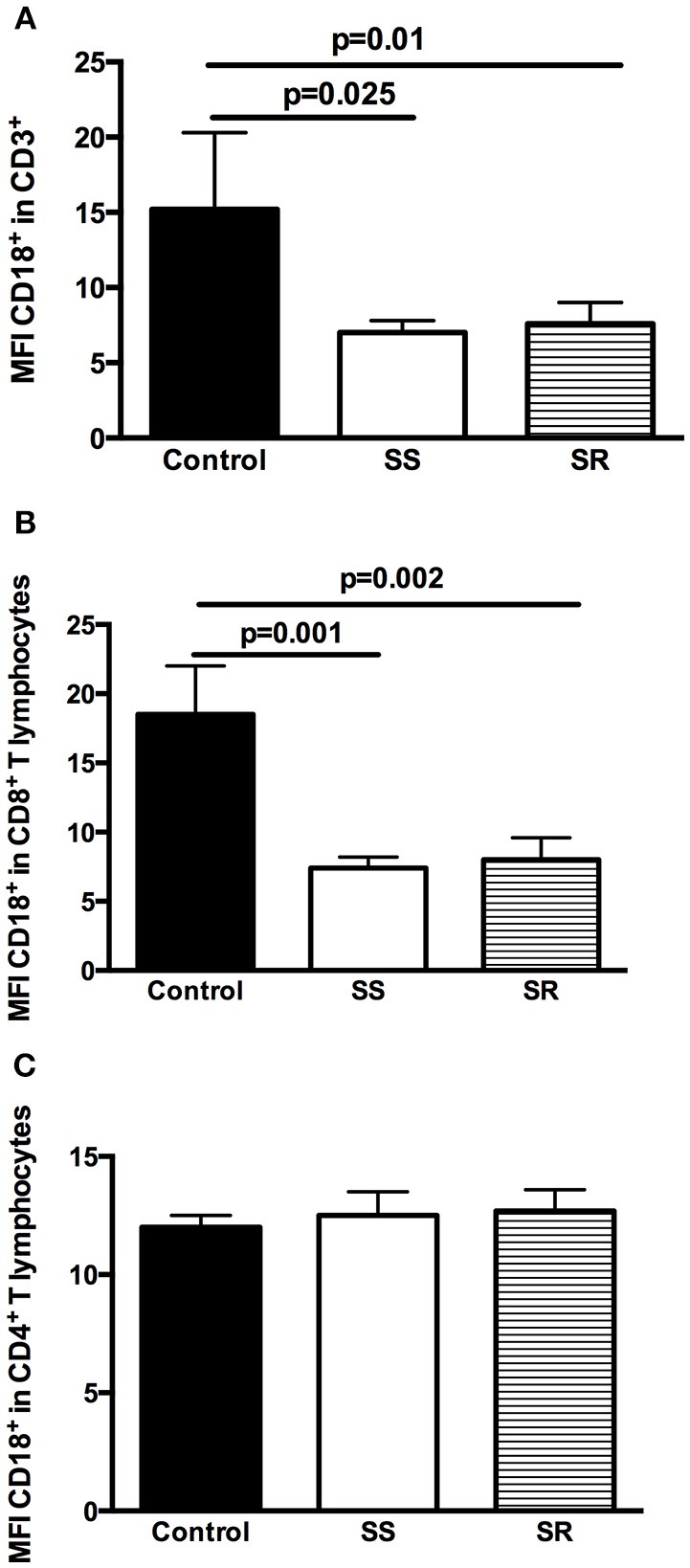
Analysis of the expression of CD18 on T lymphocytes and subpopulations in peripheral blood lymphocytes of health controls and children with INS steroid-sensitive (SS) and steroid-resistant (SR): **(A)** CD3^+^CD18^+^ lymphocytes; **(B)** CD3^+^CD8^+^CD18^+^ lymphocytes; **(C)** CD3^+^CD4^+^CD18^+^ lymphocytes. Results are expressed as bar graphs with mean values and standard deviation. *P*-values are displayed above lines that indicate each comparison.

Analysis of the percentage of T-lymphocytes (CD3^+^) expressing CD18^+^ in patients with INS revealed significantly lower percentages of this marker in SS (7.0 ± 0.8% vs. 15.2 ± 5.1% in control group, *p* = 0.025) and in SR patients (7.6 ± 1.4% vs. 15.2 ± 5.1% in control group, *p* = 0.01) in comparison to controls (Figure 3A). SS and SR patients had almost equal percentages of T-lymphocytes (CD3^+^) expressing CD18^+^ (7.0 ± 0.8% in SS vs. 7.6 ± 1.4% in SR group, *p* = 0.92, Figure 3A).

As shown in Figure 3B, similar findings were obtained in the percentage of TCD8^+^ lymphocytes expressing CD18^+^. SS and SR patients had significantly lower percentages of these cells than healthy controls (7.4 ± 0.8% in SS vs. 18.5 ± 3.5% in control group, *p* = 0.001 and 8.0 ± 2.0% in SR vs. 18.5 ± 3.5% in control group, *p* = 0.002, Figure 3B). Percentages of TCD8^+^ lymphocytes expressing CD18^+^ were similar in SS and SR patients (7.4 ± 0.8% in SS vs. 8.0 ± 2.0% in SR, *p* = 0.87, Figure 3B).

On the other hand, no significant differences were detected in the expression of CD18^+^ in TCD4^+^ lymphocytes cells in controls (12.5 ± 1.0%), SS (12.7 ± 0.5%), and SR (12.0 ± 0.9%) patients (*p* > 0.05 for all comparisons, Figure 3C).

## Discussion

In this study, INS patients had a lower percentage of circulating B-lymphocytes and NKT cells when compared to healthy controls. On the other hand, the percentage of TCD4^+^ cells expressing FoxP3^+^ was significantly higher in both SS and SR patients in comparison to controls, whereas the percentage of T-lymphocytes expressing CD18^+^ was lower in patients with INS than the control group. Comparisons between SS and SR patients also revealed a different profile of molecules expressed in immune cells. SR group had lower percentages of B lymphocyte and of T-CD4^+^ cells expressing FoxP3 and expressing CTL4A4. On the other hand, no significant differences were found between subpopulations of T-lymphocytes expressing CD18 in the comparison of SS and SR groups.

Inflammatory events seem to play a pivotal role in INS development and progression (26, 30). Particularly, the role of B-lymphocytes has been supported by the beneficial effect of B cell-associated depletion therapies, especially rituximab (29, 30). In this study, we found lower percentage of B-lymphocytes in INS patients than in healthy individuals. Our study design did not allow us to explain this finding. However, we believe that, at least in part, the previous and/or actual use of steroids might exert a long-lasting effect on B-lymphocytes. In line with this hypothesis, Baris and co-workers reported that the treatment with corticosteroids in SS patients was able to reduce B cells count to levels compared to healthy controls and the reduction persisted even after therapy cessation (31). Another possible explanation is that patients with INS in total or partial remission may have lower percentages of B-lymphocytes than healthy individuals. This possibility is corroborated by the study of Lapillonne and workers (32) in which SS patients at remission exhibited lower percentage of CD19^+^ cells (B-lymphocytes) than healthy controls (32). Interestingly, the beneficial effect of B-lymphocytes abrogation seems to be more pronounced in SS patients as shown in studies that investigated the therapeutic effect of rituximab in INS (33, 34). The percentage of circulating B-lymphocytes was significantly lower in SR if compared to SS patients. This difference might be probably related to the use of higher doses of steroids and other immunosuppressive medications in SR patients in comparison to SS group. Prospective studies with larger samples are necessary to investigate the percentage of B-lymphocytes according to disease activity and response to treatment.

Apart from B-lymphocytes, we also evaluated the percentages of NK cells, a TCD8^+^-like cytolytic innate immune cell, and of NKT cells that combine characteristics of both conventional T cells and NK cells. Compared to controls, SS and SR patients had lower percentage of NKT cells. Studies regarding the role of NKT cells in INS are limited. There is evidence that NKT cells attenuated focal and segmental glomerulosclerosis, one of the most common causes of INS, experimentally induced in mice by adriamycin administration (35). We provided first evidence for a potential role of these cells in human INS. However, further studies are necessary to investigate the effects of NKT cells.

A growing body of evidence has been pointing out to a role for NK cells in INS. Up-to-date, data reported are inconclusive and often focused in the SS population. For instance, SS patients displayed an increase in NK cells proportion during relapse, when compared with controls (36). On the other hand, an increase in NK cells was also reported in SS patients, but during remission (32). A more recent study investigated the expression of the zeta chain, a component of the T-cell receptor/CD3 (TCR) complex, and CD16 heterodimer in NK cells of the peripheral blood of INS children. These components play a crucial role in T and NK cells activation and proliferation. In comparison to healthy controls, the zeta chain expression in NK cells was lower during relapse, but not in remission of the disease. The authors suggest that the down-regulation of the zeta-chain expression in NK cells during relapse might be a sign of cell hyperactivity or merely an adaptive response to massive protein loss in the urine (18). Herein, no significant differences were found in the percentage of NK cells in the SS group in comparison to controls. However, all patients of the SS group were in remission. On the other hand, we showed that the percentage of NK cells was significantly lower in SR than in SS patients and in healthy individuals.

Over the past decades, INS has been frequently associated with excessive inflammatory response (6, 18, 26). A more recent point of view has also supported a role for immune-regulatory mechanisms in INS pathophysiology (6, 16, 17, 37–41). Pivotal cells in the regulation of the immune response are the TCD4^+^ lymphocytes expressing the transcription factor FoxP3, also known as natural T regulatory cells (T_regs_) (42). TCD4^+^FoxP3^+^ lymphocytes exert immunosuppressive effects by inhibiting the activation, proliferation and production of inflammatory mediators in several immune cells, including TCD4^+^ and TCD8^+^ lymphocytes, NK and NKT cells, B cells, macrophages and dendritic cells (43, 44). In line with this concept, we found a significant increase in the percentage of circulating T_reg_ (CD4^+^ FoxP3^+^) cells in both SS and SR group if compared to healthy controls, indicating a potential compensatory mechanism in response to the INS. Clinical and experimental studies of INS corroborate our findings, reinforcing a protective role of T_reg_ cells in INS, whereas the suppression of these cells were associated with more intense disease severity and poor prognosis (6, 16, 17, 37–41). Importantly, an increase in the proportion of circulating T_reg_ cells was found during INS remission along with an enhancement in the expression of the anti-inflammatory cytokine, interleukin-10, and the regulatory factor, transforming growth factor-β (40, 41). It is worth mentioning that there are no data regarding the role of T_reg_ cells in the differential response to steroid therapy. A single study showed that increased percentage of suppressor-inducer peripheral T cells (CD45RA^+^ CD4^+^CD25^+^) along with a low percentage of activated suppressor-effector (CD45RA^+^CD8^+^CD25^+^) might predict the likelihood of steroid sensitivity in patients with INS (45). However, the expression of FoxP3 was very rarely investigated in circulating T CD4^+^ lymphocytes. In the current study, the percentage of T_reg_ cells was higher in SS than in SR patients. In this regard, Kimata and co-workers previously reported a case of a boy with INS who achieved remission in response to influenza B virus infection and without steroid administration. Although the patient relapsed soon after remission, he was successfully treated with oral steroid therapy. Both the induction of remission and the response to steroid were associated with an increase in the number of circulating Tregs cells. The authors hypothesized that both influenza B virus infection and steroid administration increased the number of circulating Tregs, thus leading to the remission of INS (46).

The cell contact-dependent suppression by CTLA-4 (also known as CD152) is an important mechanism by which T_reg_ cells exert immunosuppression (21, 22). For instance, the expression of CTLA-4 by activated T_reg_ cells seems to downregulate CD80/86 expression on antigen presenting cells (APC). Inhibition of CD80/86 expression impairs the APC to produce the co-stimulatory signal to effector T cells via CD28, thus leading to inflammatory response (47–50). Herein, we found a significant higher percentage of circulating TCD4^+^ expressing CTLA-4 in the SS group when compared to healthy controls. Accordingly, a recent study showed increased serum levels of CTLA-4 during INS remission following prednisolone treatment, corroborating our findings (41). Of note, the authors evaluated the concentration of CTLA-4 without information regarding the type of cells expressing this molecule. In the present study, SR patients had lower percentage of TCD4^+^CTLA-4^+^ cells in comparison to SS patients and to healthy controls. Although our findings did not allow a conclusive explanation, we hypothesized that the higher expression of CTLA-4 in TCD4^+^ cells might also be associated with the successful response to steroid treatment.

LFA-1, also known as CD18, is an integrin expressed in the surface of leucocytes, crucial to the recruitment of immune cells to the injured or inflamed tissue (25). The presence of innate and adaptive immune cells, including T lymphocytes, NK cells and macrophage cells in kidney tissue has been previously described in adriamycin-induced nephrotic syndrome (51). However, it is still not known whether the recruitment of leukocytes depends on CD18 expression in patients with INS. We found lower percentages of circulating CD3^+^ lymphocytes expressing CD18 in SS and SR groups when compared to controls. Moreover, the decrease in the percentage of CD3^+^CD18^+^ cells in the periphery might reflect the recruitment of these cells to the renal tissue. Consistent with this possibility, Benz and co-workers previously found higher amounts of CD3^+^cells and macrophages in renal biopsies of patients with focal segmental glomerulosclerosis than in those with minimal change disease and in controls (16). However, CD18-associated recruitment seems to depend on lymphocyte subpopulation, since we found lower percentage of cytotoxic cells expressing CD18 in INS patients if compared to controls. In contrast, a previous experimental study of adriamycin-induced nephrotic syndrome showed that the percentage of CD8^+^ cells expressing CD18 in peripheral blood of rats increased at early stages of the disease (around 14 days) and remained elevated until later stages, around 28 days post-disease induction (51).

We are aware of several limitations of our study, including cross sectional design, relatively small sample size and inclusion of patients from a single center. The main weakness of our study was the collection of blood samples in only one occasion, which reflected just one point in time. In addition, medications in use must not be changed during blood sampling. Thus, the actual and previous use of immunosuppressive drugs may certainly interfere with measurements of subpopulations of leukocytes and it is impossible to determine the direction and intensity of this interference. The size of the control group was small due to the difficulty in recruiting healthy individuals for blood sampling simultaneously to blood collections of INS patients. Nevertheless, our study has some positive aspects including rigorous inclusion and exclusion criteria, the strict diagnosis of INS and of steroid responsiveness or not and experimental procedures performed with rigorous scientific steps.

Finally, this study found that SS INS patients have increased levels of regulatory T-lymphocytes and reduced expression of adhesion molecules. We hypothesize that differences in the profile of immune system cells might, at least in part, contribute to steroid responsiveness in SS patients. However, our findings are not conclusive and must be confirmed in large cohort studies.

## Data Availability

All the data used to support the findings of this study are available upon request.

## Ethics Statement

This study was carried out in accordance with the recommendations of Ethics Committee of Universidade Federal de Minas Gerais (UFMG), Brazil with written informed consent from all subjects. All subjects gave written informed consent in accordance with the Declaration of Helsinki. The protocol was approved by the Ethics Committee of Universidade Federal de Minas Gerais (UFMG), Brazil.

## Author Contributions

FG, RF, GB-M, WP, ER-V, and AM performed flow cytometry experiments. SP and AS collected clinical data. FG, RF, GB-M, AM, and AS analyzed flow cytometry and clinical data. FG, RF, SP, AM, and AS wrote the manuscript. AS made general supervision of all steps and submitted the manuscript. All authors approved the final version of the article.

### Conflict of Interest Statement

The authors declare that the research was conducted in the absence of any commercial or financial relationships that could be construed as a potential conflict of interest.
